# Combined laparoscopy and hysteroscopy vs. uterine curettage in the uterine artery embolization-based management of cesarean scar pregnancy: a retrospective cohort study

**DOI:** 10.1186/1472-6874-14-116

**Published:** 2014-09-24

**Authors:** Yifeng He, Xin Wu, Qiujing Zhu, Xuezhe Wu, Lingda Feng, Xia Wu, Aimin Zhao, Wen Di

**Affiliations:** Department of Obstetrics and Gynecology, Ren Ji Hospital, School of Medicine, Shanghai Jiao Tong University, 160 Pujian Road, Shanghai, 200127 China; Shanghai Key Laboratory of Gynecologic Oncology, Ren Ji Hospital, School of Medicine, Shanghai Jiao Tong University, 160 Pujian Road, Shanghai, 200127 China; Department of Gynecology, Obstetrics and Gynecology Hospital, Fudan University, 419 Fangxie Road, Shanghai, 200011 China; Department of Gynecology, First Maternity and Infant Health Hospital, Tongji University, 536 Changle Road, Shanghai, 200040 China

**Keywords:** Cesarean scar pregnancy, Methotrexate, Uterine artery embolization, Uterine curettage, Combined laparoscopy and hysteroscopy

## Abstract

**Background:**

The number of cesarean scar pregnancy (CSP) has significantly increased in the recent decade. Although uterine artery embolization (UAE) has been adopted to minimize the blood loss during uterine curettage removing of CSP, massive bleeding and uterine rupture can still be frequently encountered. The aim of this study was to compare the efficacy and safety of a novel combined laparoscopy and hysteroscopy technique with the traditional curettage in removing the conceptus and repairing the incision defect following the UAE management of CSP.

**Methods:**

The CSP patients (n = 58) diagnosed between March 1, 2005 and March 1, 2010 were enrolled in three medical centers in Shanghai, China. All of these patients have undergone intra-arterial methotrexate, UAE and one of the following treatments: combined laparoscopy and hysteroscopy (study group, n = 25) and uterine curettage (control group, n = 33). Their medical records and 2-year outcomes were reviewed. The CSP removal rate, amount of blood loss during the treatment, incision repair rate (note: the post-curettage healing process of the incision defect was seen as a form of natural incision repairing, i.e., the self-repair mode), hospital stay, β-hCG regression time and postoperative sequelae were compared between two groups.

**Results:**

The CSP removal rate in the study group (100%) was significantly higher than that (79%) in the control group (p = 0.024). The average blood loss was 78.0 mL in the study group, which was much less than the 258.5 mL (p = 0.004) in the control group. A satisfactory incision repair rate (96%) was achieved in the study group, while it was 25% (p < 0.001) in the control group. Moreover, the study group had significantly shorter hospital stays (p = 0.043) and β-hCG regression times (p = 0.033), lower rates of postoperative abdominal pain (p = 0.035) and menstruation abnormalities (p = 0.043).

**Conclusions:**

Combined laparoscopy and hysteroscopy is much safer and more effective than uterine curettage as a supplementary measure to remove the conceptus and repair the cesarean incision following the UAE management of CSP.

## Background

Caesarean scar pregnancy (CSP) is a rare type of ectopic pregnancy, which is caused by the implantation of the embryo within the prior delivery section scar [1]. Because the ectopic embryo at the scar site has extremely limited room for growth and the surrounding blood supply is relatively abundant, the clinical status of CSP is highly unstable and can progress rapidly [1]. Mothers with CSP are confronted with risks of unpredictable massive bleeding or more fatal complications, such as hemorrhagic shock and uterine rupture [1]. Although the incidence of CSP is rather low, numbering no more than 1/1,000 pregnancies, the absolute number of women suffering from this disorder has increased greatly in the past decade [1]. Especially in mainland China, CSP is no longer a rare event in clinics (increased from 7.8 cases/100,000 outpatient · years in 2000 to 36.2 cases/100,000 outpatient · years in 2010 in our institutes; data not published), which may be due to the increase of the caesarean section rate (more than 50% of all births [2]). Systemic or local administration of methotrexate (MTX) and/or uterine artery embolization (UAE) followed by uterine curettage to remove the conceptus has been suggested as standard treatment for CSP [3–6]. The primary purpose of MTX and/or UAE is to minimize blood loss during the curettage. However, these treatments do not eliminate the risk of massive bleeding or uterine perforation thereafter. According to other reports and our own experience, massive bleeding and penetrating injury to the uterus may still be encountered by clinicians who perform curettage after UAE [5, 6]. In addition, postoperative sequelae, such as abnormal uterine bleeding and dull abdominal pain, can often occur and be attributed to the unrepaired tissue defect at the scar site [7, 8].

The tissue defect, such as the sub-endometrial microtubular tract, remaining at the incision site of the uterus isthmus is the major cause of CSP [1, 9, 10]. In recent years, we have attempted to use a minimally invasive laparoscopy and hysteroscopy combination surgery following MTX and UAE to repair this local tissue defect, as well as to remove the ectopic conceptus from CSP patients safely. The present study was designed to compare the safety and efficacy of this novel therapeutic strategy with the conventional strategy (i.e., MTX + UAE + curettage) between two patient cohorts with similar clinical characteristics. The obtained data has shown both the short- and long-term effects of this novel strategy.

## Methods

### Patients

The CSP patients, who were diagnosed between March 1, 2005 and March 1, 2010 at First Maternity and Infant Health Hospital, Tongji University; Obstetrics and Gynecology Hospital, Fudan University and Ren Ji Hospital, School of Medicine, Shanghai Jiao Tong University; Shanghai, China, comprised the patient pool of this study. The research protocol was approved by the ethics committee of the three hospitals above. The inclusion criteria were (i) history of previous cesarean delivery before hospitalization, (ii) first-visit ultrasonography revealing an empty uterine and cervical canal and a myometrial defect at the caesarean scar site that was surrounded by a rich blood supply and in which a gestational sac was embedded and (iii) being immediately treated with intra-arterial MTX + UAE and followed by combined laparoscopy and hysteroscopy or curettage. The exclusion criteria were (i) receiving MTX treatment or curettage before hospitalization and/or presenting a massive uterine hemorrhage and (ii) being treated with conservative or other surgical measures, eg. intramuscular MTX, curettage, hysteroscopy, etc., as the first-line therapeutic methods. A total of 58 patients were therefore included and then divided into two cohorts (groups), i.e., 25 patients in the study group, who received intra-arterial MTX + UAE + combined laparoscopy and hysteroscopy, and 33 patients in the control group, who received intra-arterial MTX + UAE + ultrasound-guided curettage. The medical records and follow-up information of these patients were carefully and thoroughly reviewed.

### Treatment

The initial treatment for patients in both groups was the same; the right femoral artery was punctured, and 5.0-F Cobra catheters (Cordis, Brentford, Middlesex, UK) were inserted into the bilateral uterine arteries under angiographic guidance. A dose of MTX (50 mg/kg, Hengrui, Lianyungang, Jiangsu, China) was injected into each catheter, and blood flow was blocked by gelatin sponge microbeads of two sizes: 500–700 μm and 700–900 μm (Alicon, Hangzhou, Zhejiang, China). Patients were given one of the following treatments: curettage or combined laparoscopy and hysteroscopy. Both procedures were conducted by a same group of clinicians within 48 hours after UAE (we noted the hemostatic effect of UAE reached the maximum within 48 hours and declined thereafter in our previous clinical practice; data not published). (i) Uterine curettage: The internal cervical os was dilated; the conceptus was detached with a curette under ultrasound guidance and carefully pulled to the cervical canal, and then it was dragged out of the external cervical os with a vascular clamp. The curettage was terminated if the intraoperative bleeding reached 200–400 mL, and this condition was treated accordingly (e.g., vaginal gauze, uterine tamponade, intravenous injection of aminomethylbenzoic acid or reptilase). If conservative treatments failed and/or the cumulative bleeding reached 800 mL, wedge resection or hysterectomy was adopted. Laparotomy was immediately performed in cases of uterine perforation, and the uterus was either repaired or removed according to the severity of the lesion. (ii) Combined laparoscopic and hysteroscopic surgery: Laparoscopy was used for intraoperative surveillance and intra-peritoneal surgery. The vesico-uterine excavation was exposed by laparoscopy to visualize the uterine isthmus. Hysteroscopy was used to determine the exact location of the ectopic conceptus and to estimate the minimal distance from it to the uterine serosa. The conceptus was removed with a diathermy loop using the coagulating resection and tearing maneuver under the direct visualization of hysteroscopy. The local tissue weakness or defect was further evaluated based on the degree of translucency of the isthmus wall to hysteroscopic light. The defect was closed using 1–0 absorbable suture under laparoscopy (using an interrupted suturing method; generally, 1–3 stitches could be enough to close the defect). The quality of the tissue repair was evaluated by hysteroscopy. Patients in both groups received 3-day postoperative antibiotics, and the volume of vaginal bleeding and the severity of abdominal pain (mild: bearable without any sedatives; moderate: bearable with a common dose of paracetamol; severe: unbearable and requiring pethidine) were recorded. The serum β-hCG level was monitored every other day. Patients with <10 mL of daily vaginal bleeding (determined by weighting the sanitary napkins used every day) and a decrease in serum β-hCG over two consecutive tests were discharged. The main operative outcomes were divided into two types: success and failure. A successful treatment was defined as a complete/partial removal of the ectopic conceptus, cease of the abnormal vaginal bleeding and preservation of the uterus. A treatment failure was defined as one of the following conditions: ongoing growth of the ectopic conceptus, continuous vaginal bleeding and loss of the uterus.

### Follow-up

All patients had been followed up on a regular basis for two years. The initial follow-up schedule was once per week from the first day after discharge. This schedule was adjusted to once every three months if the serum β-hCG level returned to normal as demonstrated by two consecutive β-hCG tests. The follow-up items that were assessed included abdominal pain, vaginal bleeding, menstrual status and serum β-hCG level, as well as the uterine ultrasonography. All patients were advised to use contraception for at least one year. Information regarding repeat CSP and placenta implantation of the patients who became pregnant during the follow-up period was obtained by medical record review.

### Statistical analysis

The differences in categorical data between the two groups were compared using the two-sided χ^2^ test or Fisher’s exact test, as appropriate. A two-sided Student’s t test was used to compare the differences in the β-hCG level, the size of the gestational sac and the depth of implantation between the control patients with massive bleeding (>800 mL) and the remaining patients in the control group. SPSS 12.0 software (IBM, Armonk, New York, USA) was used, and p < 0.05 was considered statistically significant.

## Results

### Demographics and clinical characteristics

The demographic and clinical characteristics of the patients in the study and control groups have been listed in Table 1; there were no significant differences found between the two groups.

**Table 1:** **Comparison of the demographic and clinical characteristics between the study and control groups**

Items	Study group* (n = 25)	Control group*(n = 33)	P value**
Age (years)			0.992
≤30	5 (20)	7 (21.2)	
31-40	16 (64)	21 (63.6)	
>40	4 (16)	5 (15.2)	
Gravidity (times)			0.451
≤3	17 (68)	17 (51.5)	
4-6	7 (28)	14 (42.4)	
>6	1 (4)	2 (6.1)	
Parity (times)			0.841
1	24 (96)	32 (97)	
2	1 (4)	1 (3)	
Previous cesarean sections (times)			0.841
1	24 (96)	32 (97)	
2	1 (4)	1 (3)	
Years since the last cesarean section			0.170
≤2	3 (12)	8 (24.2)	
3-5	16 (64)	13 (39.4)	
>5	6 (24)	12 (36.4)	
Symptoms			0.316
Menolipsis only	17 (68)	17 (51.5)	
Menolipsis + vaginal bleeding	4 (16)	10 (30.3)	
Menolipsis + abdominal pain	1 (4)	0 (0)	
Menolipsis + vaginal bleeding + abdominal pain	3 (12)	6 (18.2)	
β-hCG level at hospitalization (IU/L)			0.543
≤10,000	15 (60)	15 (45.5)	
10,001-30000	7 (28)	13 (39.4)	
>30,000	3 (12)	5 (15.2)	
Diameter of the gestational sac (cm)			0.899
≤1	2 (8)	3 (9.1)	
2-3	14 (56)	20 (60.6)	
>3	9 (36)	10 (30.3)	
Minimal distance between conceptus and uterine serosa (cm)			0.278
≤0.5	5 (20)	4 (12.1)	
0.6-1	6 (24)	4 (12.1)	
>1	14 (56)	25 (75.8)	

*Data are presented as number (%).

**Two-sided χ^2^ test.

### Intraoperative bleeding, complications and hospital stay

The bilateral UAE were successfully performed in all the patients. In the study group, for the majority of the patients, laparoscopy observed an isthmus with normal appearance. Under hysteroscopy, a conceptus with a diameter of 1–6 cm (including the gestational sac, placenta, and villus), which protruded toward the uterine cavity, was found at the incision in 14 patients. In eight patients, an indentation, which contained placenta- or villous-like tissue (1–2 cm in diameter), was observed. In the remaining three patients, a conceptus (placenta, villus) with a diameter of 2–4 cm, which partially protruded into the internal cervical os and/or obstructed the cervical canal, was observed. No complications occurred during the surgeries, and the average volume of blood loss was 78.0 mL (range: 20–200 mL, Table 2). For all patients, the final hysteroscopic examination revealed a good incision repair.

**Table 2:** **Comparison of the intraoperative bleeding**, **intraoperative complications**, **and main outcomes between the study and control groups**

Items	Study group* (n = 25)	Control group* (n = 33)	P value**
Main operative outcomes			0.380
Success	25 (100)	32 (97)	
Failure	0 (0)	1 (3)	
One-time success rate^#^			0.024
Success after single surgery	25 (100)	26 (78.8)	
Success after multiple surgeries	0 (0)	7 (21.2)^##^	
Intraoperative bleeding (mL)			0.004
≤200	24 (96)	19 (57.6)	
201-400	1 (4)	9 (27.3)	
>400	0 (0)	5 (15.2)	
Preservation of fertility			0.380
Uterus intact	25 (100)	32 (97)	
Hysterectomy	0 (0)	1 (3)	
Operation time (min.)			0.354
≤60	24 (96)	32 (97)	
61-120	1 (4)	0 (0)	
>120	0 (0)	1 (3)	
Hospital stay (days)			0.043
≤5	9 (36)	6 (18.2)	
5-10	16 (64)	21 (63.6)	
>10	0 (0)	6 (18.2)	

*Data are presented as number (%).

**Two-sided χ^2^ test.

^#^The one-time success rate of the combined laparoscopic and hysteroscopic surgery or curettage after UAE.

^##^Two patients underwent massive uterine bleeding, of whom one received hysterectomy.

In the control group, the conceptus was successfully removed by a single curettage in 26 patients. The average volume of intraoperative bleeding in this group was 258.5 mL (range: 20–1600 mL, Table 2). Seven patients underwent multiple curettages because the blood loss during the first curettage reached or exceeded 400 mL. Their average blood loss was 507.5 mL (range: 200–680 mL). Of them, one patient exhibited excessive intraoperative bleeding (>800 mL), which was ceased conservatively (e.g., uterine tamponade, hemostatic drugs) in 72 hours. This patient was transfused with 400 mL of blood, and her total blood loss was 1200 mL. Another patient exhibited persistent vaginal bleeding (100–200 mL/day, which increased over time) with severe abdominal pain for two days following the curettage. After all conservative methods failed, an exploratory laparotomy was performed and revealed a perforation at the uterine isthmus. Due to the severely damaged tissue, the attempt to repair the perforation was unsuccessful, and a hysterectomy was performed. The total blood loss of this patient was 1600 mL, and the volume of blood transfused was 800 mL. The preoperative serum β-hCG levels of these two patients were 8,200 IU/L and 32,000 IU/L, respectively, which had no significant difference from that of the other patients (t = -0.774, p = 0.442, two-sided Student’s t test) of this group. Additionally, no significant differences in the size of the gestational sac (12 mm and 57 mm for the two patients, respectively) or the implantation depth (distances to the serosa were 0.7 mm and 0.5 mm for the two patients, respectively) were observed between these two patients and the other patients (for gestational sac size, t = -0.831, p = 0.409; for implantation depth, t = 1.551, p = 0.127; two-sided Student’s t test).

The average surgical time was 48 minutes (range: 35–62 minutes) in the study group and 34.6 minutes (range: 15–130 minutes) in the control group, and no significant difference was found (Table 2). The average hospital stay was 6.5 days (range: 5–9 days) in the study group and 8.7 days (range: 5–22 days) in the control group, and this difference was statistically significant (Table 2).

### Postoperative vaginal bleeding, abdominal pain, resumption of menstruation, serum β-hCG level and uterine ultrasonography

During the first three postoperative days, the daily volume of vaginal bleeding among patients in the study group did not exceed 50 mL (average: 21.9 mL/day; range: 0–50 mL/day). However, among patients in the control group it was 10–120 mL (average: 28.1 mL/day). Both the volume and duration of vaginal bleeding were significantly lower in the study group than in the control group (Table 3).

**Table 3:** **Comparison of the short**-**term symptoms and long**-**term sequelae between the study and control groups**

Items	Study group* (n = 25)	Control group* (n = 33)*	P value**
Duration of postoperative vaginal bleeding (days)			0.039
≤3	9 (36)	5 (15.2)	
4-7	9 (36)	8 (24.2)	
8-14	7 (28)	20 (60.6)	
Maximal postoperative vaginal bleeding (mL/day)			0.008
≤30	23 (92)	18 (54.5)	
31-50	2 (8)	13 (39.4)	
>50	0 (0)	2 (6.1)	
Time for serum β-hCG to return to normal (days)			0.033
≤20	19 (76)	13 (39.4)	
21-30	5 (20)	12 (36.4)	
31-40	1 (4)	5 (15.2)	
41-50	0 (0)	3 (9.1)	
Duration of postoperative abdominal pain (days)			0.572
≤3	23 (92)	32 (97)	
4-7	2 (8)	1 (3)	
Postoperative chronic pain			0.035
No pain	24 (96)	25 (75.8)	
Long-term dull abdominal pain or secondary dysmenorrhea	1 (4)	8 (24.2)	
Duration of residual conceptus tissue at the scar site (months)^#^			0.163
≤1	23 (92)	25 (78.1)	
2-12	2 (8)	7 (21.9)	
>12	0 (0)	0 (0)	
Caesarean scar condition (under ultrasonography)^#^			<0.001
Totally healed	24 (96)	8 (25.0)	
With partial dehiscence or tissue indentation	1 (4)	22 (68.8)	
With full-layer dehiscence or diverticulum formation	0 (0)	2 (6.2)	
Postoperative menstruation^#^			0.043
Returned to normal	23 (92)	20 (62.5)	
Reduced menstrual flow	1 (4)	7 (21.9)	
Increased or dripping menses	1 (4)	5 (15.6)	

*Data are presented as number (%).

**Two-sided χ^2^ test.

^#^The patient number was 32 in the control group due to one case of hysterectomy.

Mild to moderate abdominal pains were reported by all patients. The average duration of postoperative pain was similar between the two groups (Table 3). During the follow-up period, one patient in the study group and eight patients in the control group reported a recurrent dull and/or occasionally intensified pain. The difference in the rate of occurrence of such chronic pain was significant between the groups (Table 3).

Patients in both groups (except one case of hysterectomy) resumed menstruation within one month of surgery (Table 3). However, more patients in the control group reported irregular (reduced or increased) menstrual flows, elongated menstrual periods or abnormal inter-menstrual bleeding than those in the study group (Table 3).

The average serum β-hCG regression time was significantly shorter in the study group (19.6 days, range: 15–23 days) than in the control group (29.3 days, range: 17–46 days, Table 3).

A postoperative ultrasonographic evaluation was performed for all patients one month after the surgery. High-density light spots, which suggest the existence of a residual conceptus, were found in two patients in the study group and seven patients in the control group (Table 3). Expectant management was recommended to eight patients because their β-hCG levels were normal, and no signs of bleeding were found. For one patient in the control group, whose serum β-hCG level was elevated, a course of intramuscular injections of 50 mg MTX (first day) and 5 mg folic acid (second day) were applied for four cycles. Her serum β-hCG level declined to normal after one month, but at that time a small mass of conceptus could still be detected by ultrasonography.

### The two-year follow-up, ultrasonography and pregnancy outcomes

Regular uterine ultrasonography was performed for each patient. All of the residual conceptuses disappeared by the end of the follow-up. However, unhealed incision defects were observed in a few patients in both groups. Specifically, in one patient from the study group, a small submucosal dehiscence (2 × 7 × 10 mm^3^) was found. In two patients from the control group, tissue fissures (or diverticula), which reached the uterine serosa, were detected. Additionally, incision dehiscence or indentations at different degrees were found in 22 patients from the control group. Compared to the study group, the rate of incision defects was significantly higher in the control group (Table 3).

After CSP, few patients wanted to become pregnant again. Only one patient in the study group desired pregnancy. However, this patient ultimately failed to conceive. Three patients from the study group and two from the control group reported induced abortion due to unplanned pregnancies. Their treatments all went smoothly, and no cases of repeat CSP were encountered (Table 3).

## Discussion

The present study has, for the first time, comprehensively compared the efficacies of combined laparoscopy and hysteroscopy and uterine curettage as supplementary measures after UAE in the management of CSP. Our findings demonstrate that the former technique can be the superior choice. Since 1998, we have accumulated more than ten years of clinical experience in treating CSP. Worldwide, the strategies for CSP treatment have been continuously renewed [11]. More than a dozen treatment methods have been established, including the systemic MTX combined with curettage, intra-gestational injection of MTX, the transvaginal removal of the CSP, the laparoscopic removal of the CSP, and the hysteroscopy-assisted evacuation of the CSP [12–19]. These techniques were all developed to supplant the old techniques, though they have their own drawbacks, including prolonged hospital stay and extended duration of β-hCG recovery (e.g., systemic or intra-gestational injection of MTX [12, 13]), high risk of massive bleeding and loss of the uterus (e.g., uterine curettage after systemic MTX [16, 17]) and high risk of collateral injuries to adjacent organs (e.g., transvaginal removal of the CSP [18, 19]). Our experience indicates that a better treatment for CSP is the one that offers a shorter hospital stay, lower risk of severe complications, a safer surgical process, better recovery of the function of the uterus, and fewer postoperative sequelae. This study showed that the strategy of intra-arterial MTX + UAE + combined laparoscopy and hysteroscopy could satisfy these criteria. Especially for patients with active vaginal bleeding, this strategy can avoid the adverse outcomes a non-hemostasis-oriented (e.g., MTX administration) or a non-visual technique (e.g., curettage) could encounter.

Massive bleeding and uterine rupture are two severe complications of CSP [1, 12–19]. Before the introduction of UAE, the risk of uncontrollable bleeding threatened every clinician who performed curettage and/or administrated MTX [16, 20]. The incidence of massive intraoperative bleeding has reached 32%-73% [5, 6, 12]. Because only a few rigid and brittle muscle fibers exist within the scar [1, 21–24], it could be very difficult to cease the massive bleeding by traditional medical measures without UAE, even under hysteroscopy [19, 25–28]. Deans et al. have reported the use of hysteroscopy to treat six CSP patients without blocking the blood supply. As a result, one patient developed active uterine bleeding, to whom the local hemostasis was ineffective, and ergometrine and a Foley catheter tamponade were used [19]. On the other hand, because the scar tissue is extremely weak and the conceptus is very close to the uterine serosa (usually <1 mm) [25], uterine perforation or rupture can often occur as tissue necrosis emerges (e.g., after MTX treatment) or after improper surgery [19]. For instance, Dean et al. reported the occurrence of hematuria in a CSP patient after an operative hysteroscopy, suggesting the occurrence of a penetrating injury [19].

The adoption of UAE represented a technical advancement, but limitations remain. One limitation is that the independent cure rate of this technique is rather low. Among CSP patients treated with UEA, 63%-73% require curettage to resolve vaginal bleeding or to remove the unresorbable conceptus [4–6]. Another limitation is that the scar tissue can be extremely fragile after UAE, especially after co-administration of MTX [6]. Although a single dose intra-arterial infusion process can enhance the local concentration of MTX, the following necrosis of the peri-conceptus tissue and the detachment of the gestational sac can weaken the fastness of the uterine wall. The reported rate of penetrating injures or massive bleeding during curettage following intra-arterial MTX + UAE or UAE alone has been 8%-17% [4–6, 29]. In our study, 15% (5/33) of patients in the control group experienced intraoperative bleeding of >400 mL, among whom 40% (2/5) received a blood transfusion and 20% (1/5) required emergency hysterectomy. In contrast, under guided laparoscopy, a meticulous hysteroscopy can be performed, which could significantly reduce the rates of intra- and postoperative massive bleeding, uterine perforation and rupture, as was demonstrated by our cohort analysis (no cases, Table 2). Previously, Yang et al. have indicated that the risk of massive bleeding could be increased by 17 times in patients with preoperative serum β-hCG of >50,000 IU/L during the curettage [6]. Considering that β-hCG is mainly secreted by the villi and decidua, the theory of Yang et al. can be interpreted into that the implantation depth of the embryo (or the mature degree of villi and placenta) can determine the amount of intraoperative bleeding. However, in our study, the preoperative β-hCG levels of the two patients who experienced massive bleeding (>800 mL) during the curettage were both <50,000 IU/L, and there were no significant differences in the size or implantation depth of the gestational sac between these two patients and the other 31 patients in the control group. Therefore, it is highly possible that other factors influenced the volume of blood loss, and these factors might be related to the number of re-opened collateral circulations after UAE, the degree of angiogenesis around the scar site or the deformity and irregular expansion of the newly formed arteriolae [30]. Given this large number of potential factors, it is rather difficult for a clinician to predict the severity of intraoperative bleeding prior to surgery. Therefore, as a highly controllable technique, the superiority of combined laparoscopy and hysteroscopy can be significant, as was validated by our present study.

Combined laparoscopy and hysteroscopy also showed its superiority in reducing the postoperative sequelae in CSP patients (Table 3). Compared with the 22% (7/32) of control patients who had remaining conceptus tissues after the curettage, the residual rate of only 8% (2/25) among the study group is much better. The possible reasons for the high residual rate following curettage are as follows. (i) The intraoperative ultrasonography cannot detect tiny (i.e., 1–5 mm) conceptus tissues, which can continue growing or can be wrapped in blood clots, organized and enlarged several weeks later. (ii) The conceptus tissues can be flattened during the curettage, but after that, they can return to their original shapes. (iii) For a few patients, due to a tight connection with the uterus, part of the conceptus can be purposefully left to prevent massive bleeding. Unlike curettage, under direct visualization, combined laparoscopy and hysteroscopy can thoroughly remove the conceptus. This ability has also been reflected in the reduced postoperative bleeding and a more rapid regression of serum β-hCG (Table 3). In addition, patients in the study group experienced other benefits from this new technique, including (i) a lower rate of chronic postoperative abdominal pain or secondary dysmenorrhea, (ii) fewer occurrences of reduced or dripping menses and (iii) better tissue healing at the incision site. These results can be explained by the different natures of the two techniques. (i) The lesion range of curettage is much larger than that of hysteroscopy, which can lead to extensive adhesions of the uterine cavity, thus leading to a reduced menstrual flow. (ii) The tissue defect at the incision site can rarely be self-healed without a medical repair. It could be made worse by the curettage, whereby the menstrual blood could deposit and cause dull pain and dripping menses. Moreover, if endometrial cells implant into the myometrium via the defect, leading to adenomyosis, secondary dysmenorrhea may occur.

This study has some limitations. One limitation is that, as a retrospective study, the patients were not randomly allocated to the treatment arms which might have caused biases, such as the clinical observation bias occurred between medical centers and the technical selection bias for patients with different disease severities. The second limitation is that the influences of the two strategies on the future pregnancy outcomes cannot be properly compared because the desire to conceive was notably low in both groups. Thirdly, we also cannot evaluate the effect of the treatment cost on patient’s choice, as the medical record did not show any information on the treatment expense of each patient. Nevertheless, considering that the additional cost of combined laparoscopy and hysteroscopy over curettage is approximately 1,000 USD, which can be afforded by most of our patients, we believe this strategy may be a preferential choice in future gynecological practice.

## Conclusions

Combined laparoscopy and hysteroscopy, which can safely and thoroughly remove the ectopic conceptus and repair the scar tissue defect, is more suitable than uterine curettage as a supplementary measure for the UAE-based management of CSP.

## Abbreviations

CSP: Cesarean scar pregnancy
UAE: Uterine artery embolization
MTX: Methotrexate.
